# Association of *Trypanosoma vivax *in extracellular sites with central nervous system lesions and changes in cerebrospinal fluid in experimentally infected goats

**DOI:** 10.1186/1297-9716-42-63

**Published:** 2011-05-11

**Authors:** Jael S Batista, Carla MF Rodrigues, Herakles A García, Francisco SB Bezerra, Robério G Olinda, Marta MG Teixeira, Benito Soto-Blanco

**Affiliations:** 1 Department of Animal Sciences, Universidade Federal Rural do Semi-árido (UFERSA), BR 110 - Km 47, CEP: 59625-900, Mossoró-RN, Brazil; 2 Institute of Biomedical Sciences, Universidade de São Paulo (USP), São Paulo, SP, Brazil

## Abstract

Changes in cerebrospinal fluid (CSF) and anatomical and histopathological central nervous system (CNS) lesions were evaluated, and the presence of *Trypanosoma vivax *in CNS tissues was investigated through PCR. Twelve adult male goats were divided into three groups (G): G1, infected with *T. vivax *and evaluated during the acute phase; G2, infected goats evaluated during the chronic phase; and G3, consisting of non-infected goats. Each goat from G1 and G2 was infected with 1.25 × 10^5 ^trypomastigotes. Cerebrospinal fluid (CSF) analysis and investigation of *T. vivax *was performed at the 15^th ^day post-infection (dpi) in G1 goats and on the fifth day after the manifestation of nervous system infection signs in G2 goats. All goats were necropsied, and CNS fragments from G1 and G2 goats were evaluated by PCR for the determination of *T. vivax*. Hyperthermia, anemia and parasitemia were observed from the fifth dpi for G1 and G2, with the highest parasitemia peak between the seventh and 21^st ^dpi. Nervous system infection signs were observed in three G2 goats between the 30^th ^and 35^th ^dpi. CSF analysis revealed the presence of *T. vivax *for G2. Meningitis and meningoencephalitis were diagnosed in G2. PCR were positive for *T. vivax *in all the samples tested. In conclusion, *T. vivax *may reach the nervous tissue resulting in immune response from the host, which is the cause of progressive clinical and pathological manifestations of the CNS in experimentally infected goats.

## Introduction

Trypanosomes belonging to the salivary group, represented by species associated with sleeping sickness and those responsible for causing a disease known as "nagana" in ruminant livestock, cause severe economic losses in Africa, where parasite transmission occurs through the tsetse fly, a biological vector [1-3].

The clinico-pathological signs frequently reported in most outbreaks are fever, anorexia, lethargy, anemia, progressive emaciation, a rapid decline in milk production, stillborn offspring and return to estrus. Recently, Batista et al. [4] described important clinico-pathological and epidemiological aspects of natural infection by *T. vivax *that had not yet been reported in outbreaks in cattle in the Americas. Nervous signs and histological CNS lesions have been described in naturally infected cattle in Northeastern regions of Brazil, contributing to the elucidation of some pathological aspects of the CNS disease in cattle. The central neurological nature of the disease that occurs in Africa, which is caused by trypanosomosis that is triggered by salivary trypanosomes, must also be considered as an important manifestation of the disease in the Americas [5].

The presence of *T. vivax *in the CNS parenchyma, which is associated with changes and lesions in this site as described in trypanosomosis for other trypanosomes found in saliva, has not been reported. Therefore, this study was aimed at evaluating the changes in CSF, describing the anatomical and histopathological CNS lesions, and investigating the presence of the parasite in the brain of goats experimentally infected with *T. vivax *at 15 and 30 days post-infection using polymerase chain reaction (PCR).

## Materials and methods

### Experimental animals

Twelve male goats of undefined breeds, aged approximately one year, were used in the study, and the animals were housed in stalls at the Veterinary Hospital of the Universidade Federal Rural do Semi-Árido.

Ethical procedures were based on the Brazilian law 6638 (May 8, 1979) "Normas para Prática Didático-Científica da Vivissecção de Animais" and "Ethical Principles for Use of Experimental Animals" from Colégio Brasileiro de Experimentação Animal (COBEA), Brazil, which are in accordance with the "European Convention for the Protection of Vertebrate Animals used for Experimental and Other Scientific Purposes" (Strasbourg, March 18, 1986).

### *T. vivax *infection

Before being inoculated with *T. vivax*, the animals were observed for two weeks, weighed, treated with anthelmintic drugs and subjected to clinical and hematological exams. The animals were randomly selected to make up the three groups, characterized as the following: group 1 (G1), composed of four goats (nos. 1, 2, 3 and 4) infected by *T. vivax*, evaluated during the acute phase of the disease; group 2 (G2), made up of four infected goats (nos. 5, 6, 7 and 8) and evaluated during the chronic phase; and group 3 (G3), consisting of four goats (nos. 9, 10, 11 and 12) not infected by *T. vivax*.

The *T. vivax *strain used in the experiment was obtained from the blood of a parasitemic cow affected by natural infection during an outbreak in Paraíba state, northeastern Brazil [5]. The blood was collected in tubes containing 1 mg/mL of ethylenediaminetetracetic acid (EDTA), mixed with 8% glycerol and frozen in liquid nitrogen (-196°C). The isolate was thawed, and each animal from G1 and G2 was inoculated intravenously with 1 mL of blood containing approximately 1.25 × 10^5 ^*T.vivax *trypomastigotes, estimated according to the method described earlier [4].

In the three groups, daily clinical exams were performed to evaluate rectal temperature, the appearance of the visible mucosa and the volume of lymph nodes measurable by external palpation, as well as the behavior and general state of the animals. In G1 and G2, parasitemia was evaluated concurrently with each clinical examination using a thick drop of blood spread on a slide and covered by a cover slip, according to the technique described earlier [4].

### Cerebrospinal fluid (CSF) collection and analysis

CSF collections in G1 goats were performed on the 15^th ^day post-infection (dpi). In G2 goats, CSF was collected five days after the onset of nervous system infection signs. To compare the parameters, along with collections in G1 and G2, the same procedures were carried out with the animals in G3.

CSF collection was carried out with the aid of chemical tranquilization using xylazine hydrochloride at a dose of 0.1 mg/kg of body weight administered intramuscularly, followed by mechanical restraint of the animals. Cerebrospinal fluid was obtained by puncture of the cisterna magna with 25 × 8 needles and was later transferred to glass flasks. Samples of CSF were analyzed according to previously described methodology [6]. The appearance was evaluated by comparing the tube containing the sample with another tube filled with distilled water against a white surface. Densities were obtained by refractometry. The total cell count or cellularity was performed in a Neubauer chamber immediately after obtaining the samples in order to avoid cellular degeneration. Total protein and glucose values were determined using a commercial reagent set (Katal, Belo Horizonte, Brazil). The readings were performed via spectrophotometry using the biochemical analyzer Bio-2000 (Bioplus, Barueri, Brazil). In addition, an investigation of *T. vivax *was conducted in fresh CSF between a slide and coverslip by light microscopy.

### Pathological study

To carry out the anatomical and histopathological exams, all goats from G1 and two goats from G3 were euthanized on the 15^th ^dpi. Goats no. 5, 6, and 7 from G2 died spontaneously between the 35^th ^and 38^th ^dpi. The two remaining goats from G3, along with goat no. 8 from G2, which did not die spontaneously, were sacrificed on the 38^th ^dpi.

During necropsy, after the macroscopic exam, tissue fragments were collected from the CNS and were fixed in 10% buffered formalin. After fixation, the CNS was cross-sectioned at the telencephalon (frontal, parietal, occipital and temporal cortex regions), internal capsule and basal ganglia, thalamus, mesencephalon at the corpora quadrigemina, cerebelum, cerebellar peduncles, pons, medulla oblongata and the cervical, thoracic and lumbar medullae. The fixed tissues were embedded in paraffin, cut to 5 μm thickness and stained with hematoxylin and eosin (H&E).

### DNA analysis

Samples of approximately 1 cm^3 ^from the cerebral cortex, internal capsule and cerebellar white matter from G1, G2 and G3 animals were collected and placed in Eppendorf tubes containing 70% alcohol. The DNA preparations were subjected to a highly sensitive PCR assay specific for *T. vivax *standardized by Cortez et al. [7]. This PCR method targets repeated gene sequences that encode cysteine proteases (Cathepsin L) and was carried out using the oligonucleotides Tvi2 (*forward: *5' GCC ATC GCC AAG TAC CTC GCC GA 3') and DTO156 (*reverse*: 5' TTAGAATTCCCAGGAGTTCTTGATGATCCAGTA 3') as primers. The diagnosis was confirmed via PCR by amplifying a DNA fragment of about 177 bp (base pairs) that is also observed in the DNA of *T. vivax *(isolated from Pantanal), which was used as positive control. The negative controls for PCR were previously selected DNA samples from the blood of non-infected goats [7].

### Statistical analysis

For statistical analysis, a completely randomized split plot design was used. The main plot factors were the infected groups or control and the subplot was time of infection. An analysis of variance (ANOVA) was used to detect differences between treatments, followed by the use of the Tukey's test at a 5% probability level for comparison between the means.

## Results

**Figure 1:**
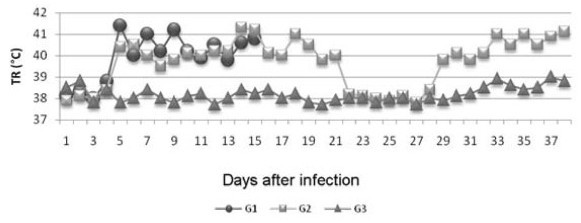
**Mean rectal temperature (RT) values of goats infected experimentally with *T. vivax *(G1 and G2) and non-infected goats (G3) as a function of the experimental period, in dpi**.

In the week that preceded infection, when the animals went through an adaptation period, no clinical abnormalities were observed. Goats from G1 and G2 showed hyperthermia on the fifth dpi, with a maximum average value of 41.3°C. In the goats from G2, this parameter was higher than average values for G3 until the 21^st ^dpi. After this period, there was a gradual reduction in rectal temperature, which remained within the normal range for the species until the 28^th ^dpi (*P *< 0.05). From the 29^th ^dpi, there was a new increase in this parameter and a significant difference in relation to G3 (Figure 1).

**Figure 2:**
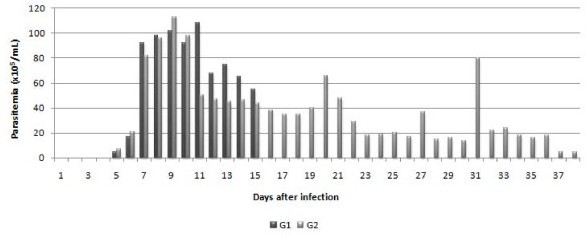
Mean parasitemia (×10^5 ^*Trypanosomas*/mL of blood) in goats infected experimentally with *T. vivax *(G1 and G2) as a function of the experimental period, in dpi.

The presence of *T. vivax *in blood was observed from the fifth dpi in G1 and G2 animals. The highest peak of parasitemia occurred from the seventh to the 21^st ^dpi (maximum of 112, 5 × 10^5 ^*Trypanosomes*/mL of blood) in G2 animals, followed by a period of low-level parasitemia between the 22^nd ^and the 31^st ^dpi. On the 32^nd ^dpi, there was a new peak of parasitemia (78.8 × 10^5 ^*Trypanosomes*/mL), followed by another reduction in parasitemia, which remained low until the end of the experimental period. The G1 animals showed similar parasitemia values to G2 animals from the first to the fifteenth dpi (Figure 2).

The goats from G2 showed markedly pale ocular, oral and preputial mucosae, an increase in lymph node volume, apathy, muscle weakness and emaciation. One goat (n. 6) had a purulent eye discharge and corneal opacity. Goats no. 6 and 8 also had dyspnea and purulent nasal mucous secretions.

Goats no. 5, 6 and 7 showed neurological disorders characterized by motor incoordination, falls, opisthotonus, nystagmus, tetany, bruxism and paddling movements on days 30, 32 and 35 post-infection (pi), respectively. The fits lasted, on average, five minutes. After this time, the animals were apparently normal and had subsequent relapses of neurological signs. Goats no. 6 and 7 had accentuated hypermetria. At the end of an average clinical course of about four days, there was worsening of the clinical status, and the animals remained in a lateral decubitus position until spontaneous death, which occurred on the 35^th ^dpi for goat no. 5, the 36^th ^dpi for goat no. 7 and the 38^th ^dpi for goat number 6, whereas goat no. 8 was sacrificed along with the two remaining goats from G3, since they did not die spontaneously due to parasitemia.

In the biochemical evaluation of CSF, G2 showed a significant increase (*P *< 0.05) in average total protein and cellularity values in addition to a significant reduction (*P *< 0.05) in the average glucose content. *T. vivax *was observed microscopically in all G2 animals. There were no significant differences (*P *> 0.05) between G1 and G3 for the CSF parameters analyzed, and *T. vivax *was not observed (Table 1).

**Table 1:** Average values of physicochemical parameters in cerebrospinal fluid taken from goats from the groups experimentally infected with *T.vivax *and evaluated in the acute and chronic phases (G1 and G2) and from goats in the control group (G3) during the experimental period.

Group	Appearance	Total protein (g/dL)	Glucose (mg/dL)	Cellularity (cell/μL)	Density (g/mL)	*T. vivax *status
G1	Clear	25^a^	74.5^a^	8.0^a^	1.006^a^	Not found
G2	Turbid	90^b^	12.0^b^	25.0^b^	1.045^b^	Found
G3	Clear	30^a^	80.0^a^	6.0^a^	1.004^a^	Not found

G1 - Group experimentally infected by *T. vivax *and evaluated in the acute phase; G2 - Group experimentally infected by *T. vivax *and evaluated in the chronic phase; G3 - non-infected group.

^a,b^Means followed by the same lower case letters in the same column are not significantly different according to the Tukey's test at a 5% significance level.

**Figure 3:**
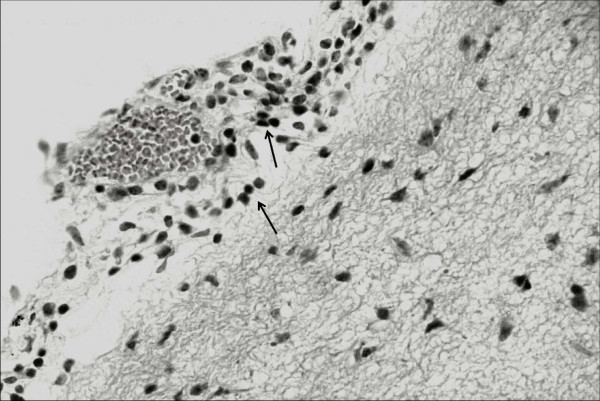
**Mononuclear meningitis (arrows) observed in goat no**. 5 from the group infected experimentally with *T. vivax *and evaluated in the chronic phase (G2). H&E, obj. 40×.

**Figure 4:**
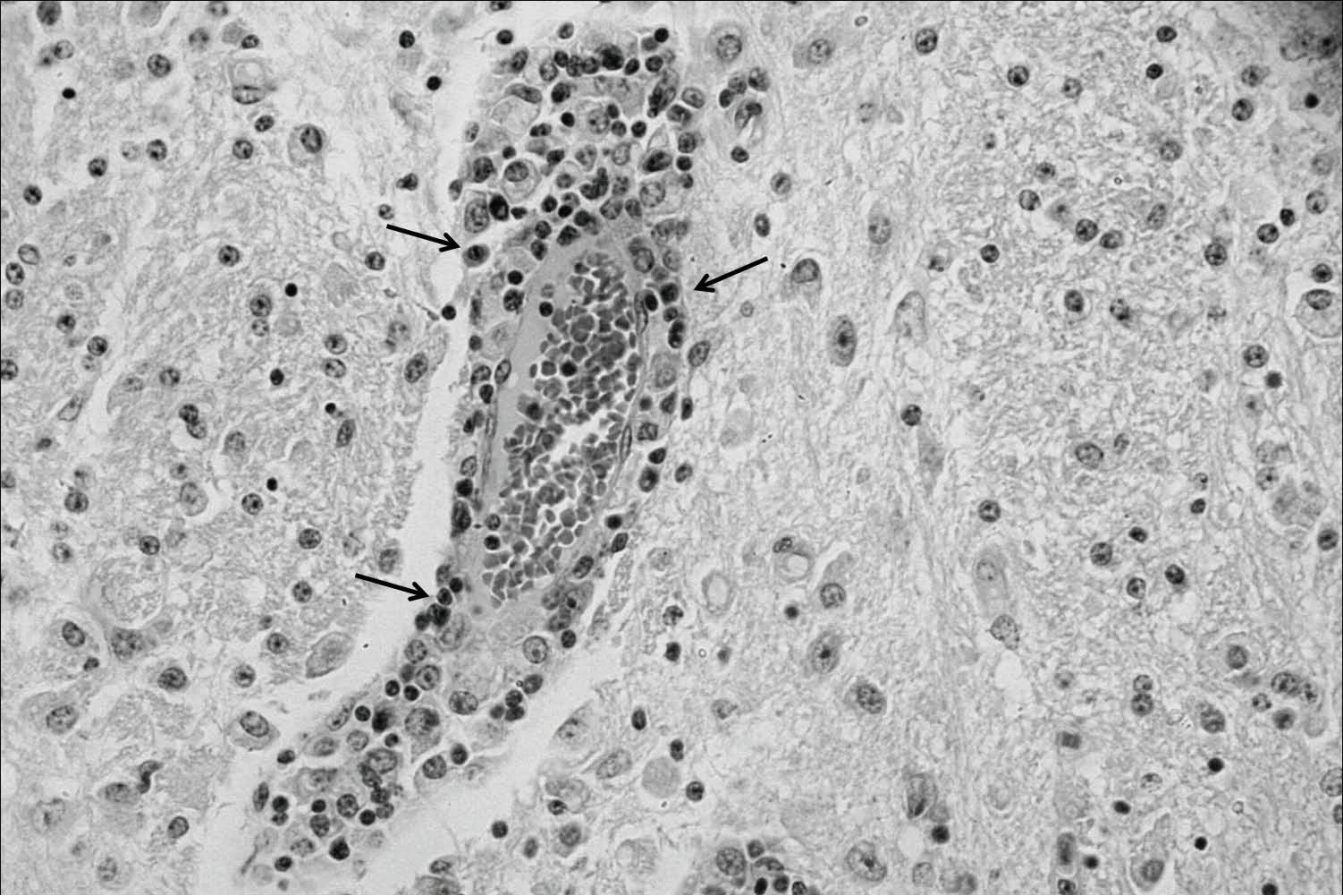
**Mononuclear perivascular inflammatory infiltrate (arrows) in the cerebellar white matter observed in goat no**. 6, which was infected experimentally with *T. vivax *and evaluated in the chronic phase (G2). H&E, obj. 40×.

In the histological exam, severe lesions were observed in the central nervous system of the goats with neurological signs. In these animals, meningitis (goat no. 5) and meningoencephalitis (goats no. 6 and 7) were observed. Meningitis was characterized by the presence of inflammatory infiltrate mainly consisting of lymphocytes and plasmocytes (Figure 3). Encephalitis was demonstrated by perivascular infiltrates composed of lymphocytes, plasmocytes and macrophages. The inflammatory perivascular infiltrates were more severe and involved a greater number of vessels in the cerebellar white matter, cerebellar peduncle and pons. In the telencephalon, at the frontal, parietal, occipital and temporal cortices, in the mesencephalon and in the thalamus, the same lesions were observed, but at a lower level of severity (Figure 4). There were no lesions in the CNS of G1 or G3 animals.

The analysis of *T. vivax *in CNS tissues by a specific PCR for *T. vivax *(TviCatL-PCR) showed the amplification of a DNA fragment of about 177 bp specific for *T. vivax *taken from the catalytic domain of the Cathepsin L gene, which was visualized in the cerebral cortex from a G1 animal. In two animals from G2, positive results were also observed in the cerebral cortex region and the cerebellum white matter. PCR was, however, negative for all G3 samples.

## Discussion

Overall, the response of the experimental infection with *T. vivax *in goats made it possible to demonstrate two clinical courses of the disease: the acute phase, which persisted for approximately two weeks and was characterized by increased hyperthermia and parasitemia, and the chronic phase or final stage of the disease, characterized by neurological disorders, which may be associated with the extravascular migration of the parasite.

Although *T. vivax *undergoes all stages of its life cycle in circulating blood, the parasite has the capacity to migrate into the tissues of a vertebrate host [8]. Thus, the participation of the parasite in the pathogenesis of inflammatory lesions that degenerate organs, such as the heart [9,10], testicle and epididymis [11] and central nervous system [4,5], has been suggested.

CSF is a biological fluid that has an intimate relationship with the central nervous system and the meninges [6,12]. The collection and analysis of CSF are of vital importance in African trypanosomosis, as they determine the clinical stage of the disease and guide the treatment method to be used in each case [13,14]. Any condition capable of affecting the meninges and the encephalon can produce inflammatory changes capable of raising the values of CSF constituents above the reference levels. As a general rule, infectious agents promote meningoencephalitis with pleocytosis and increases in total protein, which in turn modifies the appearance of CSF from clear to turbid and increases the density values [12,15]. In this study, *T. vivax *was not observed in the CSF of G1 animals; there were also no neurological signs or significant statistical differences in the physicochemical parameters of CSF when values were compared with G3 goats. As the disease progressed and the G2 goats showed neurological disorders, important changes in CSF were observed, such as turbidity, pleocytosis, high density and increased average total protein values, as well as hypoglycorrhachia. Thus, the alterations seen in the CSF from G2 goats reflect the violation of the state of health of the CNS by *T. vivax*.

Hypoglycorrhachia, the reduction of glucose values in the CSF, is generally found in hypoglycemia cases, prevention of transport through the blood-brain barrier, increases in metabolism of the brain parenchyma or infections by glycolytic organisms [15]. The CSF evaluation revealed that G2 goats showed mean CSF glucose values below the reference range for the species in addition to a significant reduction compared to values found in G1 and G3 animals [6,12]. The reduction of serum glucose levels is an important biochemical change in cattle experimentally infected with *T. vivax *and is associated with energy expenditure caused by hyperthermia and with consumption of blood glucose by trypanosomes [16].

The clinical course of the disease and the CSF changes seen in this study are also observed in humans and closely resemble the changes described in human African trypanosomosis caused by *T. brucei *[2,3]. In human African trypanosomosis, meningoencephalitis is found when cell counts exceed 5 cells/μL of CSF [17]. A similar observation was made in three G2 goats that had an average count of 25 cells/μL of CSF, coinciding with the diagnosis of meningoencephalitis subsequently proven in the histological exam. The occurrence of neurological signs associated with the presence of trypanosomes in the CSF of three G2 goats, as well as the inflammatory lesions characterized by meningoencephalitis, demonstrate the importance of *T. vivax *as the cause for clinical and pathologic manifestations of the CNS. Neurological complications associated with experimental infection by *T. vivax *were also observed by Whitelaw et al. [18], who found *T. vivax *in the CSF of goats that presented nervous system signs and meningoencephalitis.

The evaluation of goats experimentally infected with *T. vivax *isolates obtained during a cattle outbreak confirmed the tropism of *T. vivax *for the CNS. Cattle naturally infected in the outbreak mentioned above showed nervous signs characterized by incoordination, muscle tremors, opisthotonus, and hypermetria. In these animals, meningoencephalitis and malacia of the cerebellar white matter, thalamus and basal ganglia were observed [4]. The locations of brain lesions described in the goats in this study, which were more severe in the brainstem, cerebellum and meninges, were remarkably similar to those described in humans infected by *T. brucei gambiense *[17]. The clinical signs of ataxia and incoordination in the animals from G2 were consistent with cerebellar and brainstem lesions. In East African endemic areas, the rate development of nervous system signs in trypanosomosis caused by *T. brucei rhodesiense *is about 18% and generally corresponds to a diffuse meningoencephalitis with a predominance of lesions at the base of the brain [2].

The technique of DNA extraction for subsequent implementation of polymerase chain reaction (PCR), which is sensitive and specific in the detection of *T. vivax *in parasitized animals, has been refined so that not only blood [19], but also tissues [11] can be used as samples for research on the parasite. This technique has been widely employed in molecular studies because of the relative ease with which it can amplify specific in vitro regions of the genome of any organism. PCR enables the amplification of DNA sequences that are present in complex mixtures and enables a variety of studies of different natures, such as the development of highly sensitive and specific diagnosis methods, obtaining large quantities of DNA for sequencing and analyses of genetic diversity of populations.

The extravascular location of *T. vivax *plays an important role in the clarification of the pathogenesis of lesions in several systems. It is therefore notable that *T. vivax *was detected by PCR in CNS tissues for the first time, such that the parasite may be directly associated with lesions in these sites, contributing to the appearance of histopathological lesions and, consequently, to the appearance of nervous system infection signs. It is important to highlight the presence of *T. vivax *in the CNS parenchyma identified by the PCR technique in a goat from G1. This observation suggests early invasion of the CNS by the parasite, regardless of the physicochemical changes in CSF or manifestations of neurological disorders. In this way, CNS invasion by trypanosomes can be early in the infection process and may coincide with their presence in the circulation (hemolymphatic stage) [20]. Positive PCR results in nervous system areas indicate that as the disease progressed, the animals showed severe histological lesions. Therefore, the relationship between the nervous system lesions and the presence of the parasite in this site is clear.

The etiopathogenic mechanisms of CNS lesions caused by several trypanosome species remain under investigation. Some authors suggest that the lesions occur due to circulatory changes caused by emboli formed by trypanosomes, leukocytes and fibrin in capillaries and venules in the brain [21]. The immune-mediated reactions are involved in the pathogenesis of lesions, as anti-CNS antibodies are described in the CSF in infections by trypanosomes [1]. Stiles et al. [22] identified and characterized a peptide derived from *T. brucei *that induces apoptosis of vascular endothelial cells in the brain and cerebellum. Recently, Masocha et al. [23] conducted an experiment on the migration of *T. brucei *through the blood-brain barrier of rodents and concluded that while the basal membrane composition of intracerebral vessels determines the site of parasite penetration in the brain, γ-interferon is involved in the immunological control of infection, facilitating the penetration of *T. brucei *through the basal membrane of the blood-brain barrier.

In conclusion, *T. vivax *may reach the nervous tissue resulting in immune response from the host, which is the cause of progressive clinical and pathological manifestations of CNS in experimentally infected goats.

## Competing interests

The authors declare that they have no competing interests.

## Authors' contributions

JSB carried out the pathological analyses, conceived the study, and participated in its design and coordination. CMFR, HAG, FSBB and RGO carried out the experimental infection, clinical exams, and drafted the manuscript. MMGT carried out the DNA analysis. BSB participated in the design of the study, carried out the cerebrospinal fluid analysis and performed the statistical analysis. All authors read and approved the final manuscript.
